# The effects of long-term application of fomesafen on weed seedbank and resistance levels of *Amaranthus retroflexus* L.

**DOI:** 10.3389/fpls.2024.1424760

**Published:** 2024-08-13

**Authors:** Xiaotong Guo, Yulian Guo, Yu Wang, Chan Luo, Keqiang Cong

**Affiliations:** Institute of Plant Protection, Heilongjiang Academy of Agricultural Sciences, Harbin, Heilongjiang, China

**Keywords:** weed seedbank, *Amaranthus retroflexus* L., fomesafen, target resistance, non-target resistance

## Abstract

*Amaranthus retroflexus* L. is one of the invasive malignant weeds in soybean fields. Diphenyl ether herbicides are commonly used to control weeds in soybean fields currently, among which fomesafen is the most widely used. With the long-term use of fomesafen, the weed species in soybean fields in multiple areas declined, and the control effect of fomesafen against *Amaranthus retroflexus* decreased significantly. This study aims to confirm the effects of long-term use of fomesafen on weed community richness and the current resistance level of *Amaranthus retroflexus*. In this paper, the result of seed germination indicated that the weed community richness decreased significantly due to the long-term application of fomesafen, and the primary dominant weed was *Amaranthus retroflexus*. The result of the whole-plant bioassay showed that *Amaranthus retroflexus* has developed resistance to fomesafen, and the resistance index was 50~59 g a.i. ha^-1^. The resistance level and mechanism of *Amaranthus retroflexus* were clarified by target gene detection, enzyme activity determination, and gene expression analysis. The results showed that there were both target resistance with Arg128Gly mutation in the *PPX2* gene and non-target resistance (NTSR), with increased activity of metabolic enzymes (cytochromes P450 (P450s) and glutathione S-transferase (GSTs)) and protective enzymes (peroxidase (POD) and catalase (CAT)) in *Amaranthus retroflexus*.

## 1 Introduction

Cropping system diversity is currently identified as one of the five pillars of Integrated Weed Management (Riemens et al., 2022). Soil seedbank refers to the sum total of all surviving seeds and litter in the upper layer of the soil, which plays a crucial part in plant community dynamics and can affect vegetation dynamics in the short or long term (Vandvik et al., 2016; Basto et al., 2018). The weed community complex is composed of a soil seedbank and an overground weed community, which is the key to the natural continuation of weeds. Soil seedbanks can affect the occurrence, growth, and succession of overground weed communities, and are the root cause of weed harm in cropland (Buhler et al., 1997). The research of weed seedbanks has become a hot spot in weed ecology and has highly concerned scholars at home and abroad. The results indicated that weed seedbanks can be affected by weeding methods, fertilizer applications, crop rotations, and mulch, which may lead to an increase or decrease in the richness and diversity (Kaiyuan et al., 2010; Carpio et al., 2020; Auskalniene and Auskalnis, 2022). The density of weed seedbanks was different on account of the different crop rotations and tillage practices. The density of seedbanks was the highest in no-tillage continuous cropping, followed by rotation cropping, and the lowest in plowing continuous cropping (Cardina et al., 2009). The community composition can also be altered if some species are more susceptible than others due to the seed characteristics (Saatkamp et al., 2019). Although there have been many research reports on weed seedbanks, the effect of continuous application of the same herbicide on weed seedbanks is still unknown.

Nitrofen, belonging to diphenyl ether herbicides, was the first herbicide to target protoporphyrinogen oxidase (PPO, EC 1.3.3.4). Diphenyl ether herbicides are easily adsorbed in soil, with poor mobility and low leaching, and the double-ring structure can be competitively inhibited by binding to natural substrates. In recent decades, diphenyl ether herbicides have made rapid progress in the mechanism of action and product development, and many new varieties have been created and commercialized, becoming an important class of herbicides. Fomesafen, fluoroglycofen-ethyl, acifluorfen sodium, oxyfluorfen standard, and lactofen are the most widely applied in China on account of their high efficiency, wide herbicide-controlling spectrum, and low toxicity to mammals. Fomesafen is a commonly applied herbicide to control weeds in soybean fields. With the continuous and unscientific application of fomesafen, *Amaranthus retroflexus* in many regions has developed different resistance levels, resulting in a serious obstacle for soybean production (Wang et al., 2017). Therefore, it is beneficial to understand the resistance mechanism of weeds to diphenyl ether herbicides for weed control.

*Amaranthus retroflexus* L. is an invasive annual broad-leaved weed, which propagates relying on seeds. It is currently distributed in many places including Heilongjiang, Liaoning, Jilin, Inner Mongolia, Shandong, Shanxi, Henan, Hebei, and Xinjiang (Dinu et al., 2017; Qin et al., 2018). *Amaranthus retroflexus* L. is also significantly competitive in water, light, and nutrient acquisition at low density due to strong adaptability, resulting in serious crop yield reduction. Therefore, it is one of the malignant weeds in soybean, corn, vegetable, and other crop fields (Bensch et al., 2009; Sheibany et al., 2010; Ghanizadeh et al., 2014; Francischini, 2015).

PPO is the last key enzyme that inhibits protoporphyrinogen oxidase (PPO), an enzyme of chlorophyll and heme biosynthesis that catalyzes the oxidation of protoporphyrinogen IX (PPGIX) to protoporphyrin IX (PPIX). Protox inhibition leads to the accumulation of PPIX, the first light-absorbing chlorophyll precursor. PPGIX accumulation is transitory as it overflows its normal environment in the thylakoid membrane and oxidizes to PPIX (Matringe et al., 1989). Therefore, PPO can be used as the target of multiple herbicides such as diphenyl ether and cyclic imides. A large amount of protox IX is accumulated immediately when PPO is inhibited, resulting in the overflow from chloroplasts to the thylakoid membranes in the cytoplasm, and is oxidated to protoporphyrin IX by peroxidase. The accumulation of protox IX is a rapid process accompanied by the synthesis termination of chlorophyll and heme (Jacobs and Jacobs, 1993). Protoporphyrin IX, as a photosensitizer, can oxidize oxygen into toxic and highly active singlet oxygen under the light, and peroxidation occurs with the lipids of the cell membranes, resulting in membrane leakage, loss of chlorophyll and carotene, and bleaching and death of plant tissues (Lee et al., 2000).

Up to now, many weeds have developed resistance to Acetohydroxyacid synthase (AHAS) and Acetyl-CoA Carboxylase (ACCase) inhibitors. PPO inhibitors are competitive inhibitors, which can squeeze or occupy the catalytic active center of PPO to inhibit activity. There are relatively few reports of weed resistance to PPO inhibitors. In 2017, the resistance of *Amaranthus retroflexus* L. to fomesafen was first discovered in China (Heap, 2024). Agricultural production was seriously restricted by weed resistance, and the research on weed resistance has mostly focused on AHAS and ACCase, while the research on PPO is relatively scarce. The mechanism of weed resistance to PPO inhibitors needs to be further researched.

Regarding specific physiological changes, the accumulation of reactive oxygen species can be enhanced by the stress in plants (Hasanuzzaman et al., 2020; Kerchev and Breusegem, 2022). Reactive oxygen species can play a role as signaling molecules at low concentrations, while they may cause oxidative stress reactions at high concentrations (Katerova et al., 2023). Plants are exposed to many environmental factors, the effects of which cannot be predicted under practical conditions and can only be detected experimentally (Grzesiak et al., 2017; Rivero et al., 2022). Herbicides have been reported as possible causes of oxidative stress and toxicity in a variety of plants (Hasanuzzaman et al., 2020). It has been reported that the malondialdehyde (MDA) content can be increased by clodinafop in *Triticum aestivum* L. and *Secale cereale* L (Lukatkin et al., 2013). Malondialdehyde, a well-known marker of plant stress, was measured to assess the effects of herbicides in this paper. It was reported that herbicides may also cause stress responses in tolerant crops (Radchenko et al., 2021).

Peroxidase (POD) is an oxidase widely existing in various animals, plants, and microorganisms, which can eliminate the toxicity of hydrogen peroxide, phenols, and amines. It is one of the key enzymes in the enzymatic defense system of plants under stress conditions and plays a key role in removing excess free radicals and improving plant stress resistance (Weissinger et al., 2013). Catalase (CAT) is one of the key enzymes in the biological defense system, which can decompose excess hydrogen peroxide, thereby preventing cells from being poisoned by hydrogen peroxide and alleviating peroxide damage (Jomova et al., 2015). CAT is the most effective enzyme to remove hydrogen peroxide and the activity can be increased rapidly to reduce the accumulation of hydrogen peroxide and reactive oxygen species in plants (Afzal and Fakiha, 2014). The decrease of CAT activity at the middle and late stages is due to the decrease in the antioxidant system function with the aggravating stress and time extension, which leads to the accumulation of reactive oxygen species and aggravates membrane lipid peroxidation (Mandal et al., 2008). MDA is often used as an indicator of oxidative stress degree and the structural integrity of membranes at low temperatures in plants (Posmyk et al., 2005). As one of the products of membrane lipid peroxidation, the accumulation of MDA can cause damage to cells and membranes, which can be used to reflect the strength of membrane lipid peroxidation (Wu et al., 2014).

## 2 Materials and methods

### 2.1 Investigation of weed seedbank density and relative abundance in soybean fields

Nenjiang is located in the northwest of Heilongjiang Province, which belongs to the middle cold temperate zone, with a continental monsoon climate and annual average precipitation of 550-600 mm. The soil types include swamp soil, black soil, dark brown soil, and meadow soil. Soil samples were taken from Nenjiang (124°44 ‘30 “~126°49’ 30” E, 48°42 ‘35 “~51°00’ 05” N). The soil of the sampling site was black soil, under a continuous cropping system with both no-till and plowing.

The soil samples were collected from the unplowed fields when the depth of soil melting reached more than 30 cm and the weed seeds did not germinate in spring. The samples were taken from a clod 30×30×30 cm and collected from four points in each plot according to different soil layers of 0-10, 10-20, and 20-30 cm respectively. The surface area of the soil at the sampling points was 0.36 m^2^ (30 cm× 30 cm× 4 points). The seedbank was determined by the germination method. The soil samples from different layers were quantified into pots with the size of 17 cm ×17 cm ×17 cm, and each sample filled three pots (three replications and repeated three times). The pots were placed in an open greenhouse for natural growth, and the species and number of weeds were investigated 60 days after emergence.

The characteristic parameters of the weed seedbank were calculated by counting the species composition and quantity in each layer. The seedbank density (D) is the number of weed seeds per unit area. Relative abundance (RA) is half of the sum of relative density (RD) and relative frequency (RF), calculated by the formula RA= (RD+RF)/2, where RD is the proportion of a certain seed density to the total seed density, and RF is the proportion of the sample number in the total number of all samples.

### 2.2 Plant materials

We selected one susceptive population (S) and two resistant populations (R1, R2) after preliminary resistance screening. The geographic information of different populations of *Amaranthus retroflexus* is shown in **Table 1**. The nutrient soil and vermiculite were mixed in a ratio of 3:1 and placed in pots of 9 cm×9 cm×10 cm. The seeds were soaked in distilled water for 24 hours to promote germination.

**Table 1:** The geographic information collected of *Amaranthus retroflexus*.

Populations	Geographic information (Latitude and longitude)
S	Shuangxing, Lianxing Township, Nenjiang, Heihe, Heilongjiang (49.43° N, 125.39° E)
R1	Beijiang, Dashilazi, Linjiang Township, Nenjiang, Heihe, Heilongjiang (49.12° N, 125.07° E)
R2	Qingfeng, Linjiang Township, Nenjiang, Heihe, Heilongjiang (49.10° N, 125.00° E)

### 2.3 The whole-plant bioassay

Different doses of fomesafen (AS, 250 g/L, Dalian Songliao Chemical Co., Ltd.) were sprayed using an ASS-4 automatic control pesticide spraying system, equipped with a TEEJET 8002VS nozzle (Beijing Research Center for Information Technology in Agriculture) at the 4-6 leaf stage of *Amaranthus retroflexus*. The assay was repeated two times and included three replications every time. The dose settings are shown in **Table 2**. The above-ground plants were cut off and dried at 70°C until the weight was constant at 21 days after spraying. The dry weight inhibition rate was calculated using the double logistic nonlinear regression model in Sigmaplot (*v.*12.5, San Jose, CA, USA), and the GR_50_ of different populations was calculated according to the following formula.


$$Y=C+\frac{D-C}{1+{\left(X/GR_{50}\right)}^{b}}$$

**Table 2:** The doses of fomesafen.

Populations	Fomesafen (g a.i. ha^−1^)
S	0, 23.4375, 46.875, 93.75, 187.5, 375*, 750
R1, R2	0, 93.75, 187.5, 375*, 750, 1500, 3000

375* was the recommended dose.

Where *Y* is the inhibition rate of dry weight, *D* and *C* present the upper and lower limits respectively, *b* denotes the slope of the dose-response curve, *X* is the applied dose of herbicide, and GR_50_ is the fomesafen dose required to inhibit 50% of the dry weight. The resistance index was calculated by dividing the GR_50_ of the resistant population and the susceptive population to quantify the resistance level.

### 2.4 Detecting for target resistance in the *PPX* genes

The young tissues of surviving plants under fomesafen treatment (375 g a.i. ha^-1^) were collected in 1.5 mL centrifuge tubes and 10 plants from each population were stored at -80°C. The blank control (CK) plants of the S population were sampled as described above for use. The *PPX2* genes were obtained by PCR sequence amplification. The frozen leaf tissues were ground in liquid nitrogen and the RNA was extracted using Total RNA Extractor (Trizol, B511311). The cDNA was obtained by reverse transcription PCR of the extracted RNA using the Evo M-MLV One Step RT-PCR kit (AG11606). The PCR products were recovered by the SanPrep Column DNA Gel Extraction Kit (B518131) and primers (**Table 3**) were designed to amplify the sequence. The S and R amplified sequences were spliced and compared with the *PPX2* gene (MT497883.1) reported in NCBI (https://www.ncbi.nlm.nih.gov/) to determine whether mutations had occurred in *Amaranthus retroflexus*.

**Table 3:** Primer information.

primer	Base sequence 5’-3’
RT1863-P1-F	ATGGTAATTCAATCCATTACCCAC
RT1863-P1-R	AAGAGACTGACCAATTCCCTAATGA
RT1863-P2-F	TGGAGGAGAAAATGCTTCTATCAAG
RT1863-P2-R	AATTATGCGGTCTTCTCATTCATC

### 2.5 Confirmation of non-target resistance

To confirm whether there was non-target resistance of *Amaranthus retroflexus* to fomesafen, the seedlings at the 4-6 leaf stage were treated with P450 inhibitors and GST inhibitors, either alone or in combination with fomesafen. Malathion and PBO belonging to P450 inhibitors were sprayed on seedlings at 1500 g a.i. ha^-1^ 2 hours before fomesafen treatment and the GST inhibitor 4-chloro-7-nitrobenzofurazan (NBD-Cl) was applied 2 days before fomesafen treatment with a dose of 270 g a.i. ha^-1^ (Ma et al., 2016; Oliveira et al., 2017; Varanasi et al., 2018). The S population, as a control, was treated with the P450 and GST inhibitors above. The doses of fomesafen were the same as the dose setting of the whole-plant assay above (**Table 2**). The assay was repeated three times and included three replications every time. The above-ground dry weight (dried at 70°C for three days) was weighed and converted into the dry weight inhibition (%) to calculate GR_50_.

### 2.6 Determination of MDA and activity of POD, CAT, and PPO

On the basis of the results of the whole-plant bioassay, an R population with the highest resistance level was selected. The seeds were collected from individual plants after treatment with fomesafen (375 g a.i. ha^-1^) and purified by propagating for two generations. The selected susceptive and resistant populations were seeded with the method as shown in 2.2, and the seedlings were treated with fomesafen at the 3-5 leaf stage. The doses of fomesafen were 93.75 g a.i. ha^-1^ and 187.5 g a.i. ha^-1^. Each treatment was repeated three times and controlled with water. The changes in the plants were observed regularly after application, and the leaves of the surviving plants were cut off on the 1st, 3rd, 5th, 7^th^, and 14th days. The plant samples were stored at -80°C for determination of MDA content, POD activity, CAT activity, and PPO activity in the R and S plants to clarify the difference in the performance of R and S to herbicide stress and confirm whether these factors contribute to the difference in resistance between R and S.

#### 2.6.1 Determination of MDA content

Thiobarbituric acid (TBA) 0.6 g was dissolved with a small amount of 1M NaOH, and volumed with 10% trichloroacetic acid (TCA) to 1000 mL. The reaction system was composed of an enzyme solution and 0.06% TBA, sealed in a boiling water bath for 15 minutes, rapidly cooled, and centrifugated at 1500 rpm for 10 minutes. The supernatant was measured at OD_600_, OD_532_ and OD_450_. The MDA content was calculated according to the formula (μM/g FW) =*C×V/W*, where *C* stands for the MDA concentration, *V* is the volume of extracted solution, and *W* represents the sample fresh weight. The assay was repeated three times and included three replications every time.

#### 2.6.2 Detecting POD activity

Two mL 0.05 M phosphate buffer (PB) with a pH of 7.8 consisting of KH_2_PO_4_ and K_2_HPO_4_ and a small amount of quartz sand were added to a 0.5g sample to grind in an ice bath, and the homogenate was transferred into a 10mL centrifuge tube. The mortar was rinsed with 3 mL of PB, and the rinsing solution was centrifuged at 10000 rpm at 4°C for 20 minutes. The supernatant was transferred into the centrifuge tube. Furthermore, 50mL 0.1M PB with a pH of 6 and 28 μL guaiacol were added into the beaker and heated by a magnetic stirrer until completely dissolved. After cooling, 19μL of 30% H_2_O_2_ was added and mixed thoroughly. A 20μL enzyme solution and 3 mL reaction solution were added into the cuvette (zero calibration with PBS as the control), and the value was read once every 1 minute at 470nm for a total of three times. The enzyme activity was calculated in terms of change in absorbance per minute (ΔA_470_/min·mg FW). The POD activity was calculated based on the formula U/(g·min) = (*ΔA_470_* ×*Vt*)/(*W*×*Vs*×*0.01*×*t*), where *ΔA470* represents the change in absorbance during the reaction time, *W* is the sample fresh weight, *t* denotes the reaction time, *Vt* displays the total volume of extracted enzyme extract, and *Vs* is the total volume of enzyme solution used in the determination. Each treatment was repeated three times and included three replications every time.

#### 2.6.3 Detecting CAT activity

A small amount of PB with a pH of 7.8 was added to 1.0g of tissue to grind into a homogenate. The homogenate was transferred into a 10 mL centrifuge tube, and the mortar was rinsed with the buffer solution. The rinsing solution was transferred into the centrifuge tube, and centrifuged at 4 000 rpm for 15 minutes to obtain the crude extract of CAT. Furthermore, 0.3 mL of 0.1 M H_2_O_2_ was added into the centrifuge tube and the reaction solution was transferred to a cuvette quickly to measure the OD_240_. A blank control of enzyme solution inactivation was set up in the experiment. CAT activity was calculated according to the formula U/(g·min) = (*ΔA240*×*Vt*)/(*FW*×*Vs*×*0.1*×*t*). Where *ΔA_240_* represents the change in absorbance compared to the blank control, *Vt* denotes the total volume of crude enzyme extract, *Vs* is the volume of crude enzyme extract used for determination, *FW* displays the fresh weight, and *t* is the reaction time. The assay was repeated three times and included three replications every time.

#### 2.6.4 Detecting PPO activity

PPO activity was determined using a plant protoporphyrinogen oxidase (PPO) ELISA kit (HB019-Pt, Shanghai Enzyme Linked Biotechnology Co., Ltd). Each treatment was repeated three times and included three replications every time.

### 2.7 Expression of genes related to resistance

The leaf tissue was collected at 0d, 1d, 2d, and 3d after treatment with fomesafen (375 g a.i. ha^-1^) and stored at -80°C. The expression of genes related to the metabolism of fomesafen was measured in a relatively quantitative way using a LightCycler480 II (Roche, Rotkreuz, Switzerland). The genes involved were obtained from previously published transcriptome data (Guo et al., 2023). Three replications were set with a total of 11 genes to be detected, including one housekeeping gene (*Actin*). The assay was repeated three times. RNA was extracted from frozen tissues by Total RNA Extractor (Trizol, B511311), and cDNA was obtained by reverse transcription. The cDNA was diluted 10 times as a template to be detected. The reaction mixture consisted of 2X SybrGreen qPCR Master Mix (B639271, BBI, Roche), primers (F and R), ddH_2_O, and cDNA. The primer information in the experiment is shown in **Table 4**. Statistical data were analyzed by Duncan analysis in SPSS (*v.*19.0).

**Table 4:** Primer information.

Primer	Base sequence 5’-3’
Actin-qF	TGCTGGTCGTGATCTTACTG
Actin-qR	CCTCTGGGCAACGGAAT
PPX2L-F	TTCGGAGTTCTTATCCCGTCTA
PPX2L-R	GAAGGTTCGTCCTCAGTGCC
5278-F	TCATCACCGCCACCAACA
5278-R	TACGCAGACAGAAAGTCATCCA
10392-F	TCGGGTCAGATGAAGGTGG
10392-R	GAAGTTGGTGAGACAGGGATTT
18616-F	CACAACACCTCCTCTTCCTCC
18616-R	TCTTGACTCGGCTTCCACG
19481-F	ATGAGGATTTATTGAGGGTTGC
19481-R	TGAGTTGCCGTCTATCTTTCC
19725-F	TTGTCGGAGGAAGCAGAAATA
19725-R	CAGAATCGTAATTGTGCCCAT
24299-F	CTCCTGTGCTAAATCCTAATGCT
24299-R	ATCCTCGTTCACCTTCGTTG
29331-F	TTGCCTTGGGGAGGTGTATA
29331-R	GAAGGTGGAGCCCTATTGACTA
32632-F	ACCGTTACTCATTGCTTTCCG
32632-R	GATCCAACAGTCTGGCCCTTA
39222-F	TTCGGAGTTCTTATCCCGTCTA
39222-R	ACACATGTCAGATGGAGCACG

## 3 Results

### 3.1 Density and relative abundance of weed seedbanks in soybean fields

**Figure 1:**
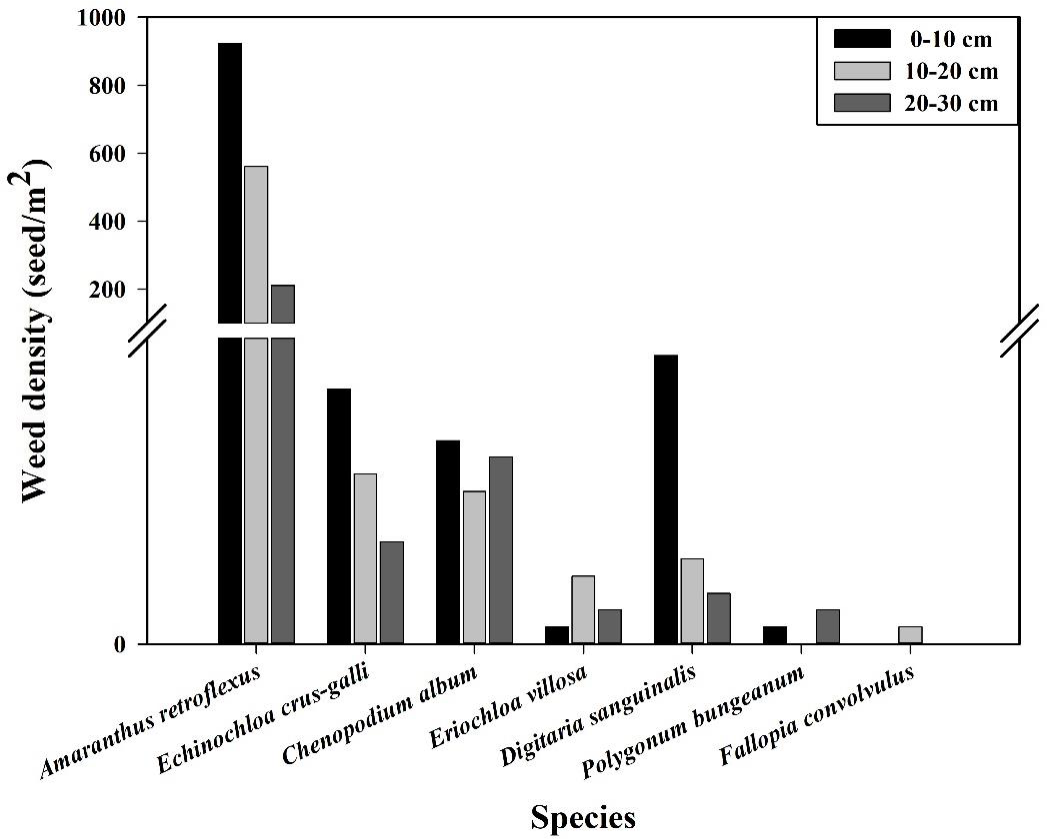
Vertical distribution of dominant weeds in the soil seedbank.

The research results of weed seedbanks in soybean fields clarified the effects of long-term stress with fomesafen on the weed seedbanks and the primary broad-leaved weed *Amaranthus retroflexus* in soybean fields. The results indicated that there were only 7 kinds of weeds in the soil, and the seedbank density of *Amaranthus retroflexus* was the highest (**Tables 5**–**7**; **Figure 1**). The species abundance of weed community was low in the 0-30 cm soil layer and primarily distributed in the 0-10cm soil layer in the continuous cropping soybean field. The total density of the weed seedbanks was 1969.444 seeds/m^2^, among which the density of *Amaranthus retroflexus* (1694.444 seeds/m^2^) was significantly higher than the sum of the other six weeds. The reason may be that the successive application of fomesafen led to an increase in *Amaranthus retroflexus* resistance year by year in soybean fields with continuous cropping for many years. Thus, *Amaranthus retroflexus* gradually became the dominant weed, affecting the occurrence of other weeds and resulting in a decrease in species abundance of weed communities in the soil.

**Table 5:** The seed amount and density of different species.

Species	Number of seeds				Density^a^ (seeds/m^2^)			
	0-10 cm	10-20 cm	20-30 cm	Total 0-30 cm	0-10 cm	10-20 cm	20-30 cm	Total 0-30 cm
*Amaranthus retroflexus*	332	202	76	610	922.222	561.111	211.111	1694.444
*Echinochloa crus-galli*	15	10	6	31	41.667	27.778	16.667	86.111
*Chenopodium album*	12	9	11	32	33.333	25.000	30.556	88.889
*Eriochloa villosa*	1	4	2	7	2.778	11.111	5.556	19.444
*Digitaria sanguinalis*	17	5	3	25	47.222	13.889	8.333	69.444
*Polygonum bungeanum*	1	0	2	3	2.778	0.000	5.556	8.333
*Fallopia convolvulus*	0	1	0	1	0.000	2.778	0.000	2.778
Total	378	231	100	709	1050.000	641.667	277.778	1969.444

a Density, the number of seeds/pots area.

**Table 6:** The number of samples germination and relative frequency of different species.

Species	Number of samples emergence				Relative frequency^a^ (RF)			
	0-10 cm	10-20 cm	20-30 cm	Total 0-30 cm	0-10 cm	10-20 cm	20-30 cm	Total 0-30 cm
*Amaranthus retroflexus*	4	4	2	10	0.364	0.308	0.167	0.278
*Echinochloa crus-galli*	4	4	4	12	0.364	0.308	0.333	0.333
*Chenopodium album*	2	2	3	7	0.182	0.154	0.250	0.194
*Eriochloa villosa*	0	1	1	2	0.000	0.012	0.083	0.056
*Digitaria sanguinalis*	1	1	1	3	0.091	0.012	0.083	0.083
*Polygonum bungeanum*	0	0	1	1	0.000	0.000	0.083	0.028
*Fallopia convolvulus*	0	1	0	1	0.000	0.012	0.000	0.028
Total	11	13	12	36	1.000	1.000	1.000	1.000

a Relative frequency, number of samples germination/total number of samples germination.

**Table 7:** The relative density and relative advantage of different species.

Species	Relative density^a^ (RD)				Relative abundance^b^ (RA)			
	0-10 cm	10-20 cm	20-30 cm	Total 0-30 cm	0-10 cm	10-20 cm	20-30 cm	Total 0-30 cm
*Amaranthus retroflexus*	0.878	0.874	0.760	0.860	0.621	0.591	0.464	0.569
*Echinochloa crus-galli*	0.040	0.043	0.060	0.044	0.202	0.176	0.197	0.188
*Chenopodium album*	0.032	0.039	0.110	0.045	0.107	0.096	0.180	0.120
*Eriochloa villosa*	0.003	0.017	0.020	0.010	0.001	0.015	0.052	0.033
*Digitaria sanguinalis*	0.045	0.022	0.030	0.035	0.068	0.017	0.057	0.059
*Polygonum bungeanum*	0.003	0.000	0.020	0.004	0.001	0.000	0.052	0.016
*Fallopia convolvulus*	0.000	0.004	0.000	0.001	0.000	0.008	0.000	0.015
Total	1.000	1.000	1.000	1.000	1.000	1.000	1.000	1.000

a Relative density, single species density/total density of all species.

b Relative abundance, RA= (RD+RF)/2.

### 3.2 The resistance of *Amaranthus retroflexus* to fomesafen

**Figure 2:**
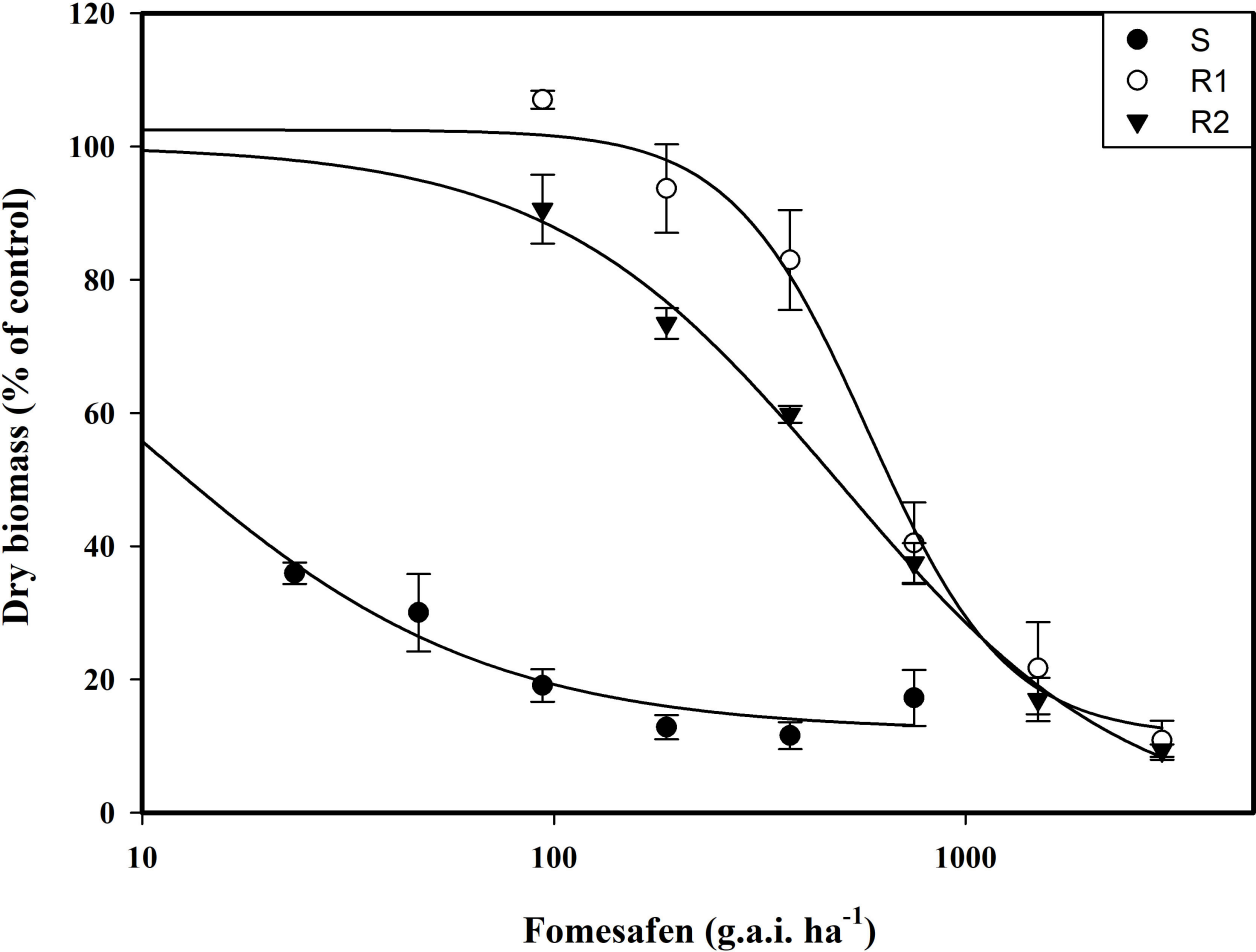
The variation of the aboveground dry weight of *Amaranthus retroflexus* populations treated with different doses of fomesafen was determined by fitting a four-parameter nonlinear regression model with a dose-response curve. Values are the mean ± SD of three biological and two technical replicates.

The resistance level of *Amaranthus retroflexus* to fomesafen was calculated (**Table 8**; **Figure 2**) based on the whole-plant bioassay. The fomesafen dose required to reduce aboveground dry weight by 50% in the S population was 9.86 g a.i. ha^-1^, while for R1 and R2 it was 583.05 g a.i. ha^-1^ and 496.73 g a.i. ha^-1^, respectively. According to GR_50_, the resistance indexes of R1 and R2 were 59 fold and 50 fold, respectively.

**Table 8:** GR_50_ and RI of different *Amaranthus retroflexus* populations treated with fomesafen.

Populations	GR_50_ ^a^ (SE)^b^ (g a.i. ha^-1^)	Correlation coefficient	Resistance index (RI)^c^
S	9.86 (4.68)	0.99	—
R1	583.05 (58.40)	0.99	59
R2	496.73 (69.26)	0.99	50

Data are the mean ± SE of two experiments, each containing three replications.

a GR_50_, the herbicide doses required in inhibiting dry weight 50%.

b SE, standard errors.

c RI = GR_50_ (R1/R2)/GR_50_ (S).

### 3.3 The Arg128 Gly mutation in the *PPX2* gene

**Figure 3:**
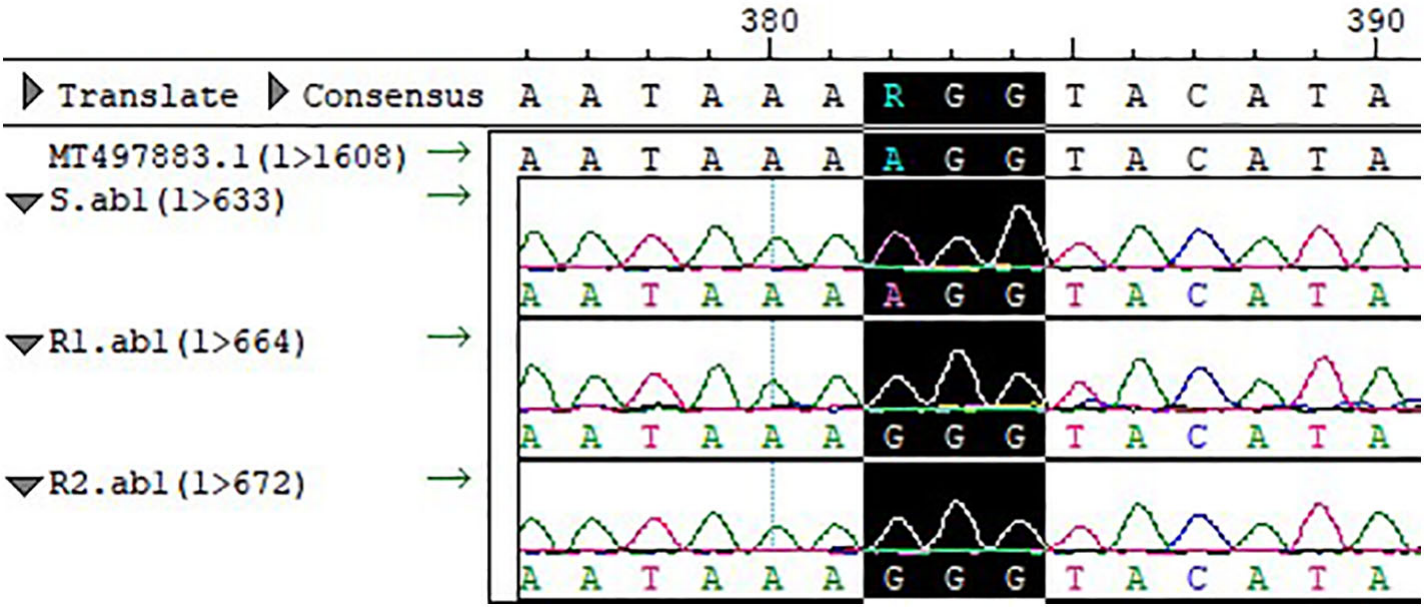
Compared with the sequence of S and template, the codon at site 128 of the *PPX2* gene of R1 and R2 was mutated from Arg to Gly (AGG→GGG).

**Figure 4:**
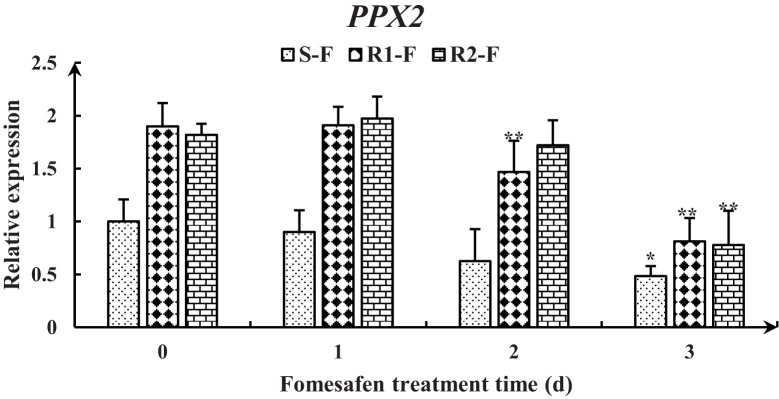
Gene expression levels of *PPX1* in the S, R1, and R2 after treatment with fomesafen are shown and were calculated using the 2^−ΔΔ^ method by real-time PCR. The Actin gene served as an internal reference gene for normalizing the variations in cDNA amounts, and the untreated populations served as a control. Values are the mean ± SD of three biological and three technical replicates. Significant differences are indicated as p< 0.05 (*) and p< 0.01 (**).

The full-length *PPX2* gene sequences of S and R were obtained by sequence amplification. The Arg128Gly (AGG→GGG) substitution (**Figure 3**) occurred in *PPX2* of R1 and R2 by comparison with the *PPX2* gene (MT497883.1) published in NCBI, and the mutation was found in all 20 single plants sequenced. The expression of the *PPX2* gene in S, R1, and R2 was analyzed by qPCR (**Figure 4**). The results indicated that there were significant differences in the expression of susceptive and resistant *PPX2* genes. This confirmed that the target resistance mechanism existed in the resistance of *Amaranthus retroflexus* to fomesafen.

### 3.4 The non-target site resistance (NTSR) of *Amaranthus retroflexus* to fomesafen

**Figure 5:**
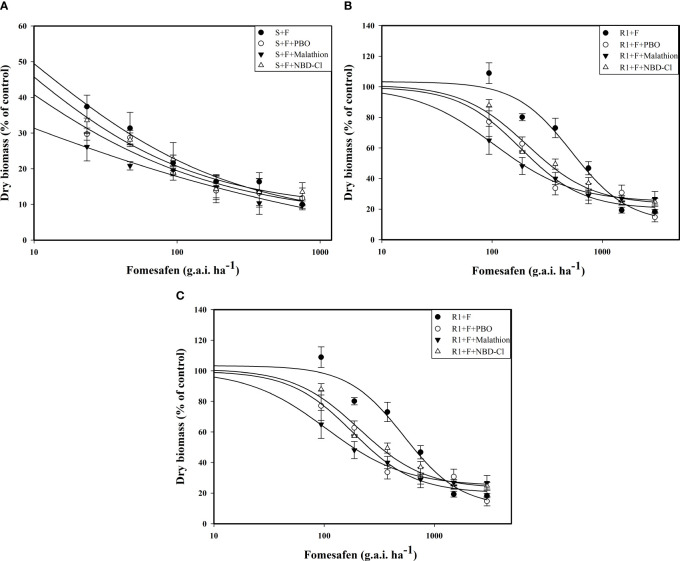
The variation of the aboveground dry weight of S **(A)**, R1 **(B)**, and R2 **(C)** treated with different doses of fomesafen (with or without PBO, malathion, and NDD-Cl) was determined by fitting a four-parameter nonlinear regression model with dose-response curve. Values are the mean ± SD of three biological and three technical replicates.

The accession of P450 and GST inhibitors resulted in a descending trend in the dose-response curve (**Figure 5**). The fomesafen dose that resulted in a 50% reduction of the aboveground dry weight of S, R1, and R2 was 8.06, 529.51, and 360.44 g a.i. ha^-1^, respectively, based on the GR_50_. With the accession of P450 and GST inhibitors (malathion, PBO, and NBD-Cl), the GR_50_ decreased significantly (**Table 9**). The control efficiency of the biomass of resistant *Amaranthus retroflexus* was increased by 34%-45% with the accession of P450 and GST inhibitors compared with the fomesafen application alone.

**Table 9:** GR_50_ of *Amaranthus retroflexus* to fomesafen with or without PBO, malathion, and NDD-Cl.

Treatments	S		R1		R2	
	GR_50_ ^a^ (g a.i. ha^-1^)	Biomass^b^	GR_50_ (g a.i. ha^-1^)	Biomass	GR_50_ (g a.i. ha^-1^)	Biomass
Fomesafen	8.06	16 b	529.51	73 b	360.44	64 b
Fomesafen + PBO	3.79	12 bc	180.32	41 cd	99.41	37 c
Fomesafen + Malathion	1.13	10 c	104.10	33 c	91.57	40 c
Fomesafen + NBD-Cl	5.59	11 c	206.50	28 d	152.90	30 c

Data are the mean ± SE of two experiments, each containing three replications.

a GR_50_, the herbicide doses required in inhibiting dry weight 50%.

b Residual biomass relative to the blank control.

### 3.5 The MDA content and activity of POD, CAT, and PPO

**Figure 6:**
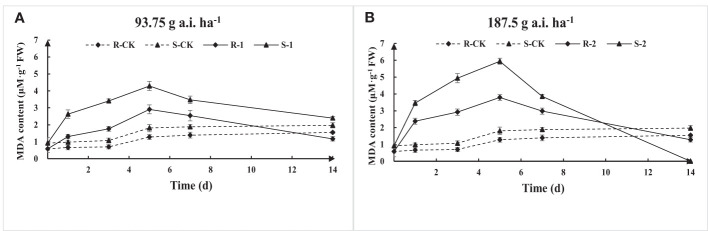
The MDA content variation in the S and R populations due to fomesafen treatment **(A, B)** over time. Values are the mean ± SD of three biological and three technical replicates.

The MDA content of S and R first increased and then decreased with the increase of fomesafen treatment time according to the results of MDA content determination (**Figure 6**). The MDA content at low-dose treatment was significantly lower than that at high doses, indicating that a high dose of fomesafen resulted in more serious damage to plant membrane lipids. The MDA content of S was significantly higher than that of R, denoting that R had stronger resistance to herbicide stress, while the membrane lipid peroxidation of S was more serious under the same treatment.

**Figure 7:**
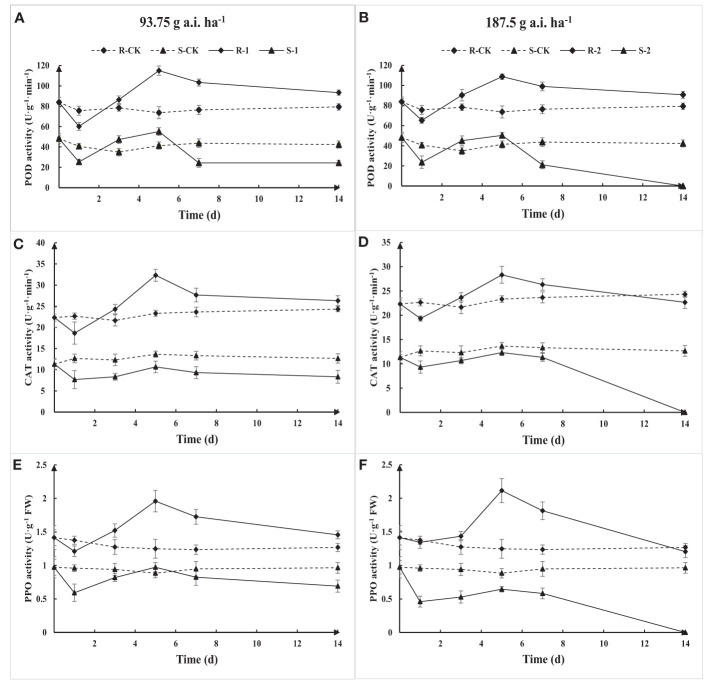
Extractable POD **(A, B)**, CAT **(C, D)**, and PPO **(E, F)** activity due to fomesafen treatment in the S and R populations over time. Values are the mean ± SD of three biological and three technical replicates.

The activity of POD, CAT, and PPO in S and R first decreased, then increased, and continued to decline with the increase of treatment time based on the determination results (**Figure 7**). The POD, CAT, and PPO activity in R gradually recovered to the initial level with the increase in application time, while the activities of S were damaged, resulting in a decrease in activity. The POD, CAT, and PPO activity in R was significantly higher than that in S under the same treatment. The results show that *Amaranthus retroflexus* can resist or reduce the herbicide stress through an increase in POD, CAT, and PPO activity.

### 3.6 The expression of related genes

**Figure 8:**
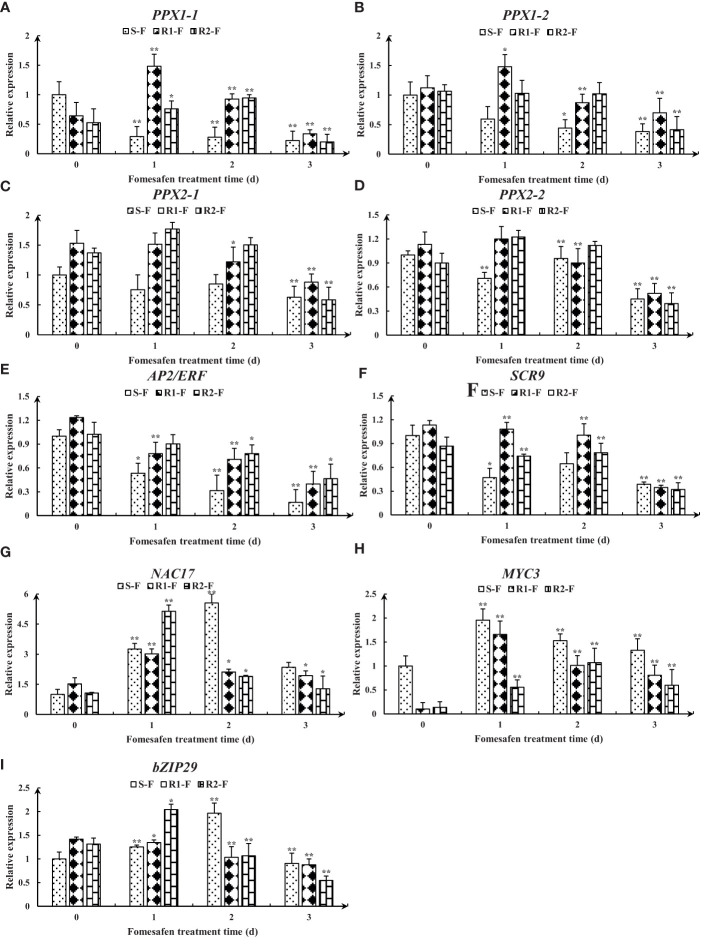
Gene expression levels of *PPX1* **(A, B)**, *PPX2* **(C, D)**, *AP2/ERF* **(E)**, *SCR* **(F)**, *NAC17* **(G)**, *MYC3* **(H)**, and *bZIP29* **(I)** in S, R1, and R2 after treatment with fomesafen are shown and were calculated using the 2^−ΔΔ^ method by real-time PCR. The *Actin* gene served as an internal reference gene for normalizing the variations in cDNA amounts, and the untreated populations served as a control. Values are the mean ± SD of three biological and three technical replicates. Significant differences are indicated as p< 0.05 (*) and p< 0.01 (**).

The nine genes were obtained from published transcriptomes (Guo et al., 2023) and their relative expression was measured by quantitative real-time PCR (**Figure 8**). The results showed that the expression of genes encoding *PPX1* and *PPX2* in R (R1, R2) first increased and then decreased, and the expression was higher than in S. The expression of the *AP2/ERF* and *SCR* genes in the TF family was decreased, but the expression in R was higher than in S. The expression of the NAC17, MYC3, and bZIP29 genes in the TF family first increased and then decreased, and the expression in R was higher than in S at 2-3 days after fomesafen application. Based on the gene expression results, we speculated that the above genes may have had an effect on the resistance of S and R.

## 4 Discussion

Changes in cropping systems can affect the depth of weed seeds in the soil and weed species, thus affecting the effectiveness of weed control. Some studies have found that crop rotation has a greater impact on seed density than tillage, and seed density generally decreases with the increase in tillage intensity (Cardina et al., 2002). It has been reported that mulch may have an effect on the weed seedbank and can be used to control weeds by combining it with the cropping system (Adeux et al., 2023). The adaptability of plant seeds may be reduced on account of climate change (Steane, 2015). Climate change has resulted in range variation in some species and even extinctions in some cases (Dullinger et al., 2012; Nogués-Bravo et al., 2018). Some plant populations will decline in the ecosystem due to a decrease in adaptability (Wambugu et al., 2023). In addition to the above cropping systems and climate change that can affect weed seedbanks, we found that the use of chemical herbicides can also have an effect on weed seedbanks. In this study, due to the long-term application of fomesafen, the *Amaranthus retroflexus* resistant to it has become the dominant weed, which seriously affected the richness of the weed seedbank. Therefore, weed seedbanks can be regulated to reduce the accumulation of weed seeds in the field by paying attention to climate change and combining planting systems with the rational use of herbicides. At the same time, agroecological weed management needs to reduce the dependence of cropping systems on herbicides, so as to achieve the sustainable management of weeds in farmland (Petit et al., 2018).

The target resistance of weeds is primarily due to the structural changes of target proteins, which affect the binding of target proteins with herbicides. The gene encoding the target enzyme PPO is located in mitochondria and plastid PPX1 and PPX2, but many current reports of resistant weeds are related to the PPX2 subunit (Dayan et al., 2017). The resistance of *Amaranthus palmeri* S. Watson to PPO inhibitors was caused by a glycine deletion at the 210th site (ΔG210). The deletion mostly existed in the *PPX2* gene and had dual targeting effects on mitochondria and chloroplasts, and it may also be the reason why fewer weed species are currently resistant to PPO inhibitors (Watanabe et al., 2001; Jung et al., 2004; Patzoldt et al., 2006; Varanasi et al., 2015). Gly210 can cover the PPO enzyme α-8 helix, and ΔG210 shortens the helix and increases the active site space by approximately half (Gressel and Levy, 2006). Sensitivity to diphenyl ether herbicides was decreased by changing the structure of the PPO enzyme and reducing the catalytic rate (Gressel and Levy, 2006; Patzoldt et al., 2006). Currently, it is deemed that the mutations of the target gene can affect the binding between target proteins and herbicides by changing its structure, resulting in weed resistance. *Ambrosia artemisiifolia* L. and *Amaranthus palmeri* were resistant to PPO inhibitors due to the Arg128Leu substitution in the PPX2 subunit, which is located in a highly conserved region of the PPO gene, and the probability of substitution is equal in the *PPX1* and *PPX2* genes (Koch et al., 2004; Chandi et al., 2012; Dayan et al., 2017; Giacomini et al., 2017). In this study, the resistance of *Amaranthus retroflexus* to fomesafen was associated with the occurrence of an Arg128Gly mutation in *PPX2*. It has also been reported that the resistance of *Amaranthus palmeri* to PPO inhibitors was caused by a Gly399Ala mutation in the PPX2 subunit [34]. The resistance levels and cross-resistance may be influenced by different mutations in the target. The target of most herbicides is quite specific, and the target gene sequence can be obtained by methods of gene cloning and sequencing analysis, and the target mutation can be detected. Compared to target resistance, non-target resistance involves a variety of complex metabolic processes, including the interaction of multiple metabolic enzymes. The non-target resistance mechanism is the reduction of the effective ingredients of herbicide at the target site, including the reduction of herbicide absorption or transport by weeds, the enhancement of the ability of metabolic detoxification of weeds to herbicides, and the shielding effect of herbicides, so as to reduce the damage of herbicides to weeds. Among these, the enhancement of the metabolic detoxification ability of weeds to herbicides is the most common mechanism (Délye, 2013; Yu and Powles, 2014). Currently, there are relatively few reports on the non-target resistance of weeds to PPO inhibitors compared to target resistance. It has been reported that the non-target resistance of *Amaranthus palmeri* to fomesafen was endowed by the metabolism enhancement of GSTs and P450s (Varanasi et al., 2018). The non-target resistance caused by metabolic enzymes was found in herbicide-resistant *Debregeasia orientalis* C. J. Chen, *Papaver somniferum* L., *Raphanus raphanistrum* L., *Ambrosia artemisiifolia* L., *Amaranthus palmeri*, and *Lolium perenne* L (Nakka et al., 2017; Torra et al., 2017; Figueiredo et al., 2018; Chahal et al., 2019; Dücker et al., 2019; Lu et al., 2019). Reduced transport of herbicides to weed target sites is also a common metabolic resistance mechanism. The resistance of *Raphanus raphanistrum* L. was caused by reduced transport of the herbicide to the target site within the weed (Goggin et al., 2016). In this paper, there was target resistance endowed by the Arg128Gly mutation in the *PPX2* gene and differences in *PPX2* gene expression. Furthermore, there was metabolic resistance caused by metabolic enzymes (P450s and GSTs) and protective enzymes (POD and CAT). There were also differences in the expression of genes encoding *PPX1* and *PPX2* and related genes of transcription families, which may cause differences in resistance levels among different *Amaranthus retroflexus* populations. In this study, metabolic enzymes, P450s and GSTs, and protective enzymes, POD and CAT, all affected the fomesafen resistance level, which also indicated that there was non-target resistance. Although single-gene inherited non-target resistance has also been reported in some weeds, non-target resistance involving detoxification enzymes is usually regulated by multiple genes, and different weeds have different detoxification regulatory genes and modes for herbicides with different mechanisms of action. These may cause the same weed to become resistant to other herbicides with completely different mechanisms of action (Preston, 2003; Délye et al., 2013). Therefore, research on non-target resistance is conducive to elucidating the resistance genes of weeds to different herbicides with different mechanisms of action and studying weed resistance to different herbicides from different perspectives such as enzymology, molecule, and metabolism.

Weed resistance is not only related to changes at the molecular level, including gene mutation and gene expression, but also at the physiological and biochemical levels. Plants produce reactive oxygen species (ROS) in normal metabolism, and the toxic effect of ROS is mitigated due to the low-level balance between the production and removal of ROS by the plant itself (Janků et al., 2019). The balance between plant ROS production and removal is broken by stress, which leads to the accumulation of free radicals and membrane damage (Zhi-Hui et al., 2007). The protective enzyme system can alleviate ROS damage caused by stress to a certain extent. Studies have indicated that oxidative stress caused by prometryn can significantly change the activity of wheat reactive oxygen scavengers, including CAT and POD, to alleviate the oxidative damage caused by environmental stress (Jiang and Yang, 2009). Therefore, their structure and activity or content levels reflect the ability of plants to resist stress to a certain extent. The content of some antioxidant enzymes in *Echinochloa phyllopogon* (stapf) Koss. that was extremely sensitive to quinclorac was lower than that in rice plants resistant to quinclorac, thus, strong antioxidant capacity was one of the reasons for the resistance development (Sunohara and Matsumoto, 2004). Under fomesafen stress, the POD and CAT activity of resistant *Amaranthus retroflexus* was significantly higher than that of susceptive *Amaranthus retroflexu*.

The development of weed resistance in soybean fields is not only related to gene mutations, but also related to crop planting patterns and irrational use of herbicides. In order to improve the yield and quality of crops, it is necessary to use the integrated weed management (IWM) strategy which combines agricultural and chemical measures to control grass. This could include, for example, the rotation of different crops and delaying planting in order to control initial weeds with non-selective herbicides. Herbicides should be applied correctly according to the application instructions, and rotation or reasonable mixing should be used to delay the development of weed resistance. At the same time, regularly observe the results of herbicide application, closely monitor problem areas where weeds are difficult to control or overgrown, identify and record the number of surviving weeds, and check for resistant weeds in order to understand any trends or changes in the weed population that exist. With the continuous development of resistant weeds caused by mutation or adaptive evolution to herbicide stress, it is more difficult to control weeds in soybean fields. The development of new varieties of diphenyl ether herbicides with high efficiency, low toxicity, environmentally friendly characteristics, and good selectivity is the primary direction. Improving the resistance detection and analysis method and understanding the resistance mechanism are conducive to the development of mixed preparations of diphenyl ether herbicides with other herbicides, so as to delay the development of weed resistance.

## 5 Conclusion

In this study, *Amaranthus retroflexus* gradually became the dominant weed due to the long-term application of fomesafen affecting the occurrence of other weeds and resulting in a decrease of species abundance in weed communities in the soil. We observed both target resistance with the Arg128Gly mutation in the *PPX2* gene, and non-target site resistance (NTSR) with increased activity of metabolic enzymes (P450s and GSTs) and protective enzymes (POD and CAT) in *Amaranthus retroflexus*. Compared to target resistance, non-target resistance poses a greater threat to integrated weed management. Non-targeted resistance may make weeds resistant to herbicides with different mechanisms of action, and even to herbicides that are not yet on the market. Understanding the resistance level and mechanism of resistant biotypes is crucial for customizing effective weed management strategies.

## Data availability statement

The original contributions presented in the study are includedin the article/supplementary material. Further inquiries can be directed to the corresponding author.

## Author contributions

XG: Writing – review & editing, Writing – original draft, Visualization, Validation, Supervision, Software, Resources, Project administration, Methodology, Investigation, Funding acquisition, Formal analysis, Data curation, Conceptualization. YG: Conceptualization, Data curation, Funding acquisition, Investigation, Project administration, Resources, Writing – review & editing. YW: Writing – original draft, Investigation, Data curation. CL: Writing – review & editing, Investigation. KC: Writing – review & editing, Investigation.
